# Giant Lipoma on the Left Shoulder: A Case Report

**DOI:** 10.7759/cureus.63067

**Published:** 2024-06-24

**Authors:** Frida L Sanchez, Ricardo J Arreola Peralta, Susana Sanchez Alvarez, Nalleli Y Boyso Suarez, Jose G Escamilla Cázares

**Affiliations:** 1 Surgery, Hospital General Regional 1, Morelia, MEX; 2 General Surgery, Hospital General Regional 1, Morelia, MEX; 3 Internal Medicine, Instituto Mexicano del Seguro Social (IMSS), Mexico City, MEX

**Keywords:** intermuscular lipoma, intramuscular lipoma, recurrent lipoma, giant lipoma resection, giant lipoma

## Abstract

A lipoma is a tumor of adipose tissue cells that can develop anywhere in the body, usually at the subcutaneous level without invading adjacent structures. Its most common location is on the back and extremities. According to the literature, it is considered common in both sexes; however, it is mentioned that it occurs more frequently in females. Clinically, it presents as a slow-growing, painless mass. Although the diagnosis is clinical, an imaging study is usually performed for confirmation and to obtain information about its location, relationship with adjacent structures, and surgical planning. The definitive treatment involves surgical resection with histopathological analysis, providing a definitive diagnosis to rule out malignancy. In the present case report, we will present a 45-year-old female patient, with no significant medical history, who comes for evaluation due to a tumor in her right shoulder approximately 5 cm in size with gradual growth to 20 cm over 12 years, seeking evaluation due to clinical manifestations. A surgical protocol was initiated, with magnetic resonance imaging (MRI) considered the imaging study of choice according to the literature, where a superficial location was observed. A surgical plan was made that included complete resection and histopathological analysis, with post-surgical evaluations at two, four, and six months post-surgery showing no signs of recurrence, remission of symptoms of nerve compression, and appropriate wound healing.

## Introduction

Lipomas commonly manifest as benign tumors located in the subcutaneous layer of mesenchymal adipose tissue. Nevertheless, a minority of cases may present in deeper anatomical sites beneath the fascia, encompassing intramuscular, intermuscular, intrathoracic, and retroperitoneal regions [1]. Benign adipose tissue tumors can be classified according to histopathological reports, with classical lipomas being the most frequent [1]. These lipomas are categorized by their location as either superficial or deep [1]. Superficial lipomas are the most common, typically appearing as solitary in 80% of cases, growing slowly, well-circumscribed, and measuring less than 5 cm in diameter [1]. Between 5% and 7% of these superficial lipomas may present as multiple tumors [1].

On the other hand, deep lipomas are larger tumors and are more vascularized, potentially invading vessels and muscles, and even enveloping nerves. Generally, lipomas predominantly appear in adults between 40 and 60 years of age, progressing slowly, and their main indication for surgical resection is either the patient's desire or the presence of clinical manifestations, such as pain and, less frequently, compression of nearby soft tissues, entrapment of peripheral nerves in close proximity, or neurological deficits arising from nerve impingement [1]. The surgical approach of choice involves addressing the lesion site with dissection of adjacent structures until complete resection [1,2].

We present the case of a 45-year-old female with a slow-growing tumor on the left shoulder, without involvement of the supraspinatus muscle and deltoid. The performed diagnostic protocol, surgical management, and histopathological analysis confirmed the diagnosis of lipoma.

## Case presentation

**Figure 1:**
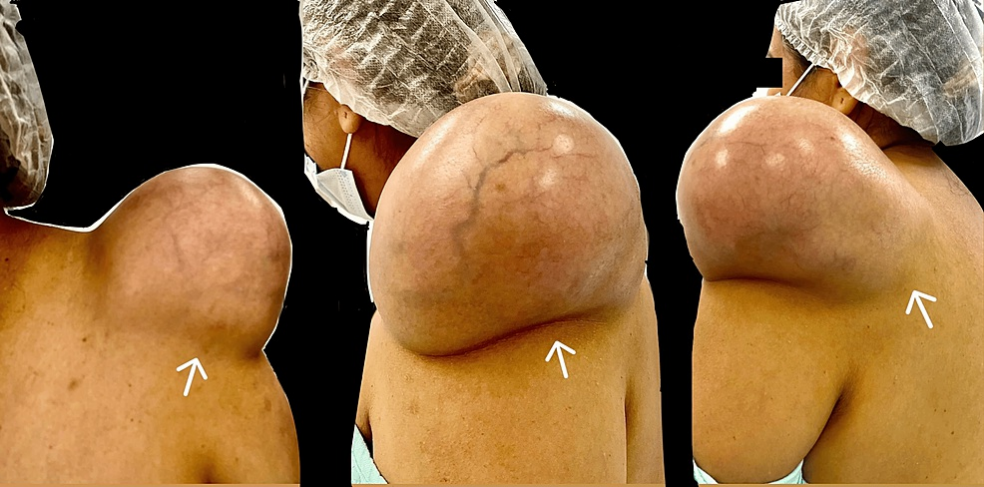
Tumor on the left shoulder The arrow shows a tumor of 20 cm in length × 15 cm in diameter × 15 cm in height, with visible vascularization and skin without alterations.

**Figure 2:**
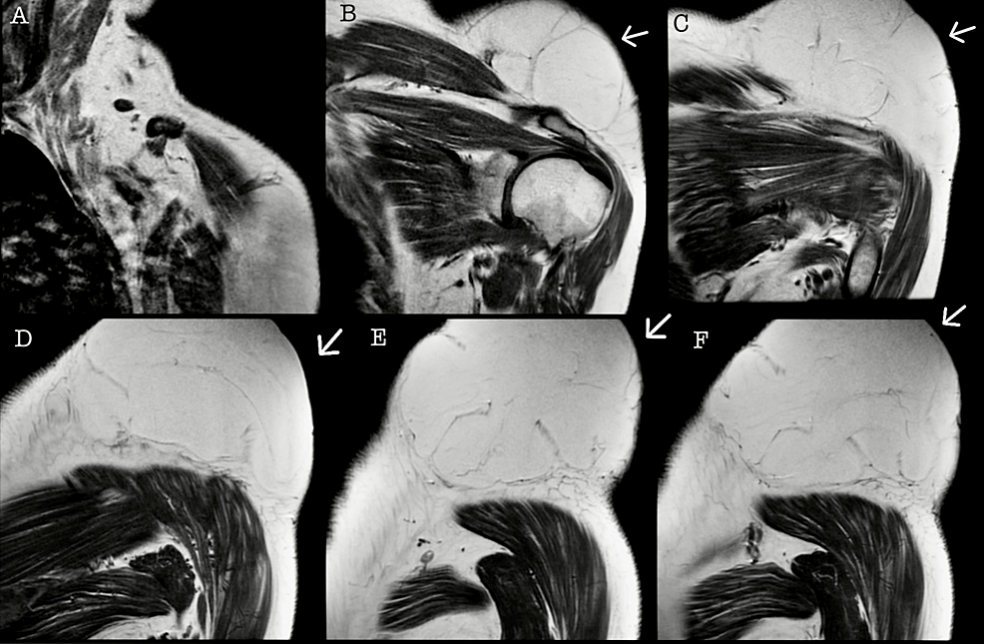
Magnetic resonance imaging in coronal section in T1 The arrow shows a hyperintense, well-defined, extrafascial tumor, without signs of bone erosion or muscular infiltration and joint involvement.

**Figure 3:**
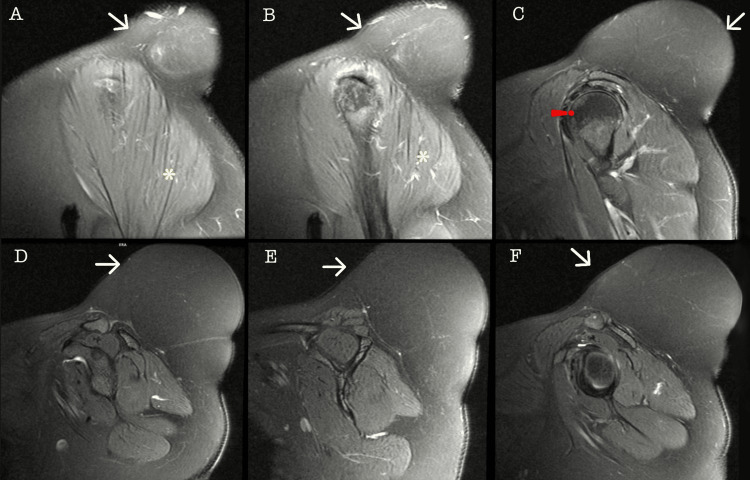
Magnetic resonance imaging in coronal section in T2 The white arrow indicates a hypointense tumor, which is compared to muscle and isointense to fat. The red line delineates the humeral head, while the yellow asterisk denotes the deltoid muscle.

A 45-year-old female with no relevant family history, chronic illnesses, allergies, or surgical history had reported the presence of a tumor in the left shoulder diagnosed in 2012, approximately 4 × 3 cm in size, painless, not adherent to deep layers, and with no limitation in arm mobility. Surgical management was offered; however, due to the absence of symptoms, the patient declined surgical treatment and was scheduled for annual appointments to monitor the tumor's progression. In August 2023, she sought evaluation in the general surgery service due to intermittent shoulder pain that increased with physical activity and decreased with rest, as well as intermittent paresthesia and limited mobility in the upper limb, partially improving with analgesics. During the physical examination, a tumor approximately 20 × 15 × 15 cm in size was observed, painful upon mobilization, not adherent to deep layers, and without skin lesions but vascularized (Figure 1). Therefore, pre-surgical studies were requested, revealing no abnormalities, and a magnetic resonance imaging (MRI) scan was conducted for adequate surgical planning and to assess muscle, nerve, or bone invasion due to the current size. In September 2023, she returned with a complete pre-surgical protocol and an MRI study of the left shoulder (Figures 2, 3).

**Figure 4:**
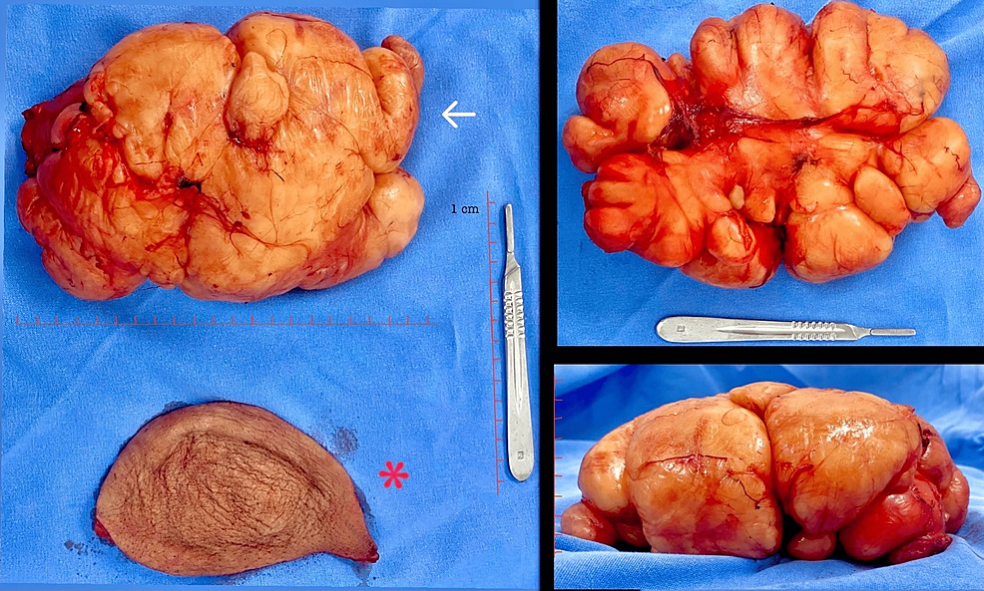
Complete lipoma resection The white arrow shows a macroscopic image of the tumor in the left shoulder, measuring 20 × 15 cm, yellowish, solid, and smooth, with complete resection, vascularization, and no presence of calcifications; the red asterisk indicates the resection of redundant skin.

**Figure 5:**
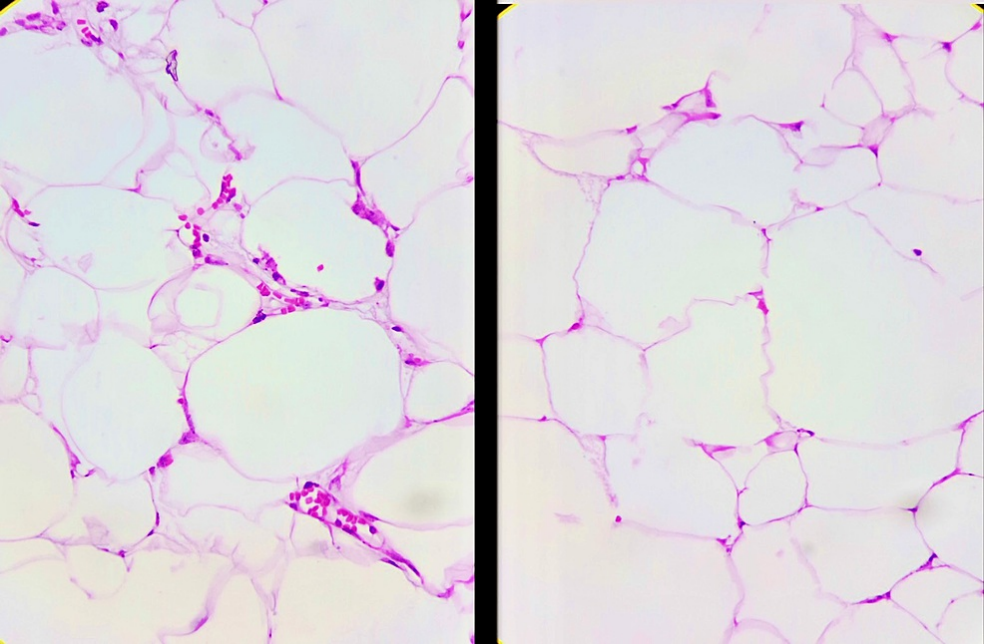
Histological sections stained with hematoxylin and eosin (40×) Mature adipose proliferation without signs of atypia or malignancy.

**Figure 6:**
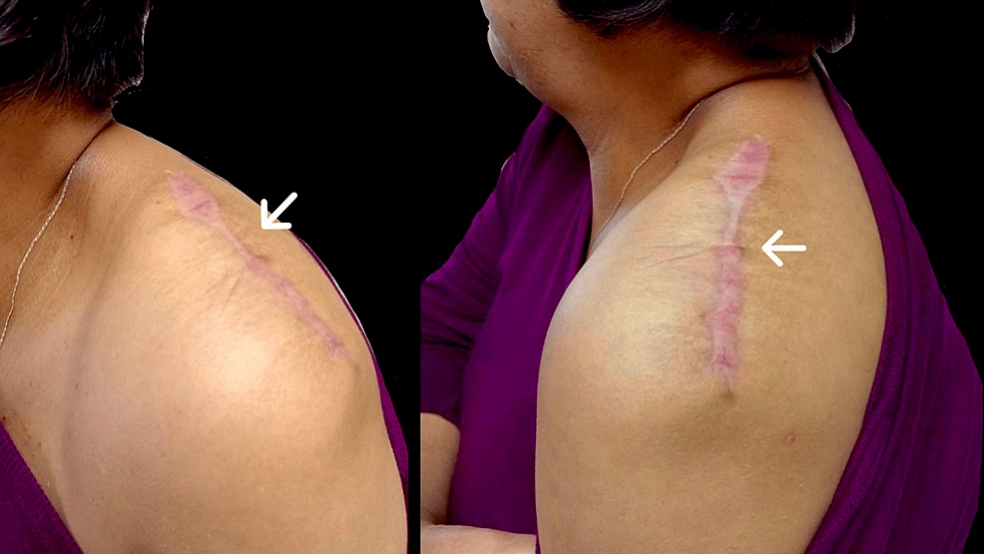
Wound six months after resection The white arrow indicates the scar after six months of surgery with no signs of recurrence.

An elective surgery was scheduled with balanced general anesthesia and multimodal analgesia, including an interscalene block for localization. A complete resection of the tumor was performed, revealing no involvement of nervous or vascular structures and no localization of a major nutrient vessel (Figure 4). No drains were placed, and the postoperative recovery was adequate. The patient was discharged without complications on the second postoperative day, with follow-up in the general surgery outpatient clinic. During the post-surgical evaluation, the pathological result was presented, reporting a "20 cm lipoma, completely resected, without signs of malignancy" (Figure 5). Outpatient appointments were scheduled monthly for the third and sixth months (Figure 6) to ensure an adequate healing process, without recurrence, no limitations in arm mobility, and no paralysis or numbness.

## Discussion

Lipomas are the most frequent soft tissue tumors in the body, able to occur in any location [1]. Benign lipomatous tumors comprise a wide and complex variety of lesions, including the classic lipoma (mature white fat cells) and its variants (angiolipoma, myolipoma, ossifying lipoma, chondroid lipoma, spindle cell lipoma, and pleomorphic lipoma), lipoblastomas (immature fat cells), hibernoma (mature brown fat cells), and infiltrating lipomas, among which lipomatosis is included [2].

Their etiology is unknown; various theories have been proposed, none of which has been fully accepted: sporadic, genetic, endocrine/metabolic, and traumatic [1]. They mostly occur in adults, and their incidence increases with age. They are generally diagnosed in the fifth and sixth decades of life, rarely occurring during childhood [2].

According to the reviewed literature, they may have a similar incidence in males and females; however, they are reported more frequently in females, as they have a higher proportion of body adipose tissue [1,3,4]. Other risk factors for lipoma development include obesity [2].

The most common locations are the trunk, neck, and proximal extremities. Locations in the cephalic extremity, hands, and feet are rare [5,6].

Clinical manifestations depend on their location, and for the most part, they appear in the subcutaneous plane and are asymptomatic [5]. Patients often report the presence of painless or minimally bothersome tumorations of variable size and slow or no growth, which can reach up to 20 cm, although in 80% of cases, they do not exceed 5 cm [2,5]. Rarely do they occur in deep planes such as intramuscular, intermuscular, periosteal, or intraosseous, and they may produce compression of adjacent nerves [1].

The possible compression of adjacent peripheral nerves by a lipoma should be considered, as well as the variables of recurrence risk, infiltration, deep localization, and various clinical and pathological manifestations. There are four conditions in which lipomas can cause compression of a peripheral nerve: solitary lipomas, which can generate nerve compression; encapsulated lipomas, which can be located in a nerve as an intrinsic lesion; lipofibromatous hamartoma, where there is a fibrofatty mass within the nerve; and macrodystrophia lipomatosa, which causes overgrowth of the extremities, especially the hands [5].

Conducting a clinical history and physical examination can lead to the diagnosis; however, imaging studies are performed to assess the extension and involvement of structures, as well as for planning surgical management. Simple X-rays reveal a radiolucent mass of soft tissues, with signs of ossification or calcification (depending on the time of evolution) [2]. Ultrasound helps detect and identify space-occupying lesions with isoechoic appearance, without shadowing, and without the presence of flow with color Doppler. Additionally, it helps differentiate between a solid and a cystic mass [1]. Magnetic resonance imaging precisely locates the tumor, its size, and limits, as well as its relationship with adjacent structures such as vessels, nerves, and muscles [1,2]. It also aids in the differential diagnosis between benign and malignant tumors [2]. The definitive diagnosis is a histological study, which rules out malignancy, such as mesenchymal tumors and liposarcoma [1,3].

Macroscopically, lipomas are well-defined masses of adipose tissue, surrounded by a thin fibrous capsule. Microscopically, they are organized into lobules of mature univacuolated adipocytes with eccentric nuclei, but without atypia, and are separated by fine connective tissue septa. Fat necrosis can be observed in larger tumors, and skeletal muscle fibers are infiltrated in intramuscular lipomas [5].

The surgical treatment involves the complete removal of the tumor [1,2]. For small or superficial lipomas, surveillance and monitoring of size can be considered; however, if there is an increase in size or discomfort, it indicates definitive surgical management [3].

## Conclusions

Lipomas are the most common soft tissue tumors and should be considered in the differential diagnosis during a general surgery consultation when a slow-growing tumor without associated symptoms is present. The size of the tumor increases the possibility of invasion of underlying structures such as muscles, nerves, or bone structures. Generally, the most frequent are classic lipomas, which have a benign etiology. Although the diagnosis is usually clinical, we can rely on magnetic resonance imaging (MRI), which is considered the study of choice during the diagnostic approach, primarily useful for planning definitive treatment. According to the reviewed literature, the preferred surgical management is complete resection, followed by histopathological analysis, to rule out malignancy. Scheduled follow-up appointments are important for evaluating patients who do not initially opt for surgical management, to assess growth and rule out infiltration into adjacent structures, as well as the onset of clinical manifestations. In the reported case, conservative management was initially considered by the patient due to the absence of symptoms, with annual monitoring until limitation in the mobility of the upper extremity associated with paresthesias appeared over a period of 12 years.
